# Analysis of Hybrid Buffering and Retransmission in OBS Networks

**DOI:** 10.1155/2015/159245

**Published:** 2015-08-18

**Authors:** D. Veera Vanitha, M. Sabrigiriraj

**Affiliations:** ^1^ECE Department, Faculty of Engineering, Avinashilingam Institute for Home Science and Higher Education for Women, Coimbatore 641108, India; ^2^ECE Department, SVS College of Engineering, Coimbatore 642109, India

## Abstract

Burst contention is a major problem in the Optical Burst Switching (OBS) networks. Due to inadequate contention resolution techniques, the burst loss is prominent in OBS. In order to resolve contention fiber delay lines, wavelength converters, deflection routing, burst segmentation, and retransmission are used. Each one has its own limitations. In this paper, a new hybrid scheme is proposed which combines buffering and retransmission, which increases the mean number of bursts processed in the system. In this hybrid method, retransmission with controllable arrival and uncontrollable arrival is analyzed. Normally all the bursts reach the first hop and few of them go for second hop to reach destination. After all the bursts reach the destination the server may go for maintenance activity or wait for the arrival of next burst. We model it as a batch arrival single server retrial queue with buffer. Numerical results are analyzed to show the mean number of bursts processed in the system with uncontrollable arrival and controllable arrivals.

## 1. Introduction 

A major problem in Optical Burst Switching [1] networks is wavelength contention which is the main reason for burst loss. Contention occurs when more than one data burst try to reserve the same wavelength channel on an outgoing link. Hence, one of the contending data burst is allowed to reserve the channel and other bursts are blocked. A research in the area of wavelength contention in Optical Burst Switching networks classifies the contention resolution methods into (a) Optical Buffering with Fiber Delay Lines (FDLs) [2]; FDLs are used to store bursts temporarily for short period of time until output ports become available. Due to limited storage time it is not possible to store a large number of optical bursts in the buffer and this follows only first in first out method. Random access of data is not possible. (b) Wavelength conversion [3] avoids contention by converting the burst from its own wavelength to the other freely available wavelengths and it improves the performance considerably. However it is expensive because it requires extra hardware. (c) Burst segmentation [4] is a burst divided into segments. Each segment may consist of a single packet or multiple packets and define the possible portioning points of a burst when the burst conflicts with other bursts. When contention occurs, only those segments of a given burst that overlap with segments of another burst will be dropped. (d) Deflection routing [5, 6]: when a burst arrives at an OBS core node, if the primary path is not available it will be switched to an alternate path and the burst is distributed to its adjacent nodes. Overall link utilization and network performance are improved greatly. It is usually more attractive than Fiber Delay Lines and wavelength conversion schemes because it requires no extra hardware cost. The performance depends on network topology and is effective only when there is small number of alternate paths. At heavy load, burst loss probability becomes large. (e) Methods based on traffic engineering like retransmission [7]: dropped burst is retransmitted again from the source. Combining two or more of the above techniques gives better performance [8, 9]. In this paper, the combination of buffering and burst retransmission in OBS networks is investigated. The main aim is to achieve the moderate end to end delay and to maximize the mean number of bursts processed in the system.

The paper is organised as follows.  Section 2 explains about the hybrid method which combines buffering and burst retransmission for two hops with a numerical example and in Section 3 numerical results are given.  Section 4 is ended with the conclusion.

## 2. Hybrid Buffering and Retransmission in OBS Networks

### 2.1. Method Analysed

In Optical Burst Switching networks the packets arrive in batches called bursts and are allowed to enter into the source. Bursts are allowed to have maximum of two hops between the source and destination. If the server is free, one of the arriving bursts reserves the wavelength and others join the buffer. Retransmission from the buffer takes place after some time. Mean number of bursts processed in the system is calculated. This paper deals with combined buffering with retransmission, controllable arrival, and maintenance activity.

**Figure 1:**
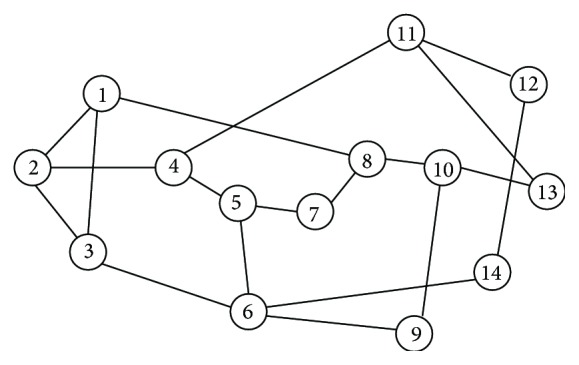
NSFnet topology.

In Figure 1, assume the source destination pairs for the bursts are (1, 3) and (1, 6). Few bursts have source destination pair as (1, 3) and the path is 1-3 (number of hops is 1). For another few bursts source destination pair is (1, 6) and the selected path is 1-3-6 (number of hops are 2). In this case the link 1-3 is called first hop (FH) for all the bursts and for remaining few bursts 3-6 is called second hop (SH). When contention occurs either in source itself or in FH the bursts are stored in buffer and retransmission from the buffer takes place. Otherwise the normal transmission process continues. After the burst reaches the destination, the server which is the data channel may go for maintenance activity or wait for the next arriving burst in the system.

### 2.2. Numerical Example

Consider an example from Figure 1 with node 1 as source and node 6 as destination to explain briefly about the end to end delay occurring in (i) normal transmission, (ii) deflection routing, (iii) pure retransmission, (iv) deflection routing and retransmission, and (v) the proposed hybrid method. The total transfer delay of a Burst Header Packet (BHP) is explained in these five different cases. Assuming the propagation delay for all links is equal then

(1) $$\begin{array}{c} T=nt, \end{array}$$

where *T* = total transfer delay of a BHP, *n* = number of hops in the path, *t* = sum of propagation delay and BHP processing time on one hop, and x = contention.

**Figure 2:**
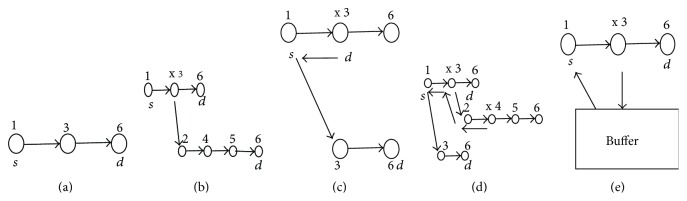
Case (a): normal transmission. Case (b): deflection routing. Case (c): retransmission. Case (d): deflection and retransmission. Case (e): proposed method.

From Figure 2 it is observed that in case (a), the burst is transmitted successfully; *T*_a_ is 2*t* where *n* = 2. In case (b) burst contention or link failure occurs and the burst is deflected through alternate path available; *T*_b_ is 5*t* where *n* = 5. In case (c) retransmission takes place; *T*_c_ is 3*t* where *n* = 3. In case (d) combination of deflection and retransmission takes place; *T*_d_ is 8*t* where *n* = 8. In case (e) due to proposed hybrid buffering and retransmission the delay *T*_e_ is 5*t* where *n* = 5 which is similar to deflection method. Though the end to end delay in this hybrid method is high compared with pure retransmission method this becomes efficient when number of hops between source and destination is large. And also in retransmission the complexity is high by keeping copies of the transmitted data. *T*_a_, *T*_b_, *T*_c_, *T*_d_, and *T*_e_ are end to end delay of cases (a), (b), (c), (d), and (e), respectively. So from the above example it is proved that the proposed hybrid buffering and retransmission method gives lower end to end delay when compared with all the above other methods.

### 2.3. System Model

A mathematical model [10] is analyzed for the proposed hybrid scheme over Optical Burst Switching networks consisting of OBS nodes connected with unidirectional links. Batch arrival retransmission queue with second service, feedback, admission control, and vacation concept is used here. This model is for the single-server infinite queuing system with buffer. Contention resolution schemes performance is based on the network traffic load. The model is developed only when number of hops between source and destination is two. Number of wavelengths used is limited to one. So no wavelength conversion is used here.

Consider a single server with two-phase retransmission. The bursts arrive in batches of variable size according to Poisson process with rate *λ* bursts/second. The batch size *Y* is a random variable with distribution function *P*(*Y* = *k*) = *C*_*k*_,  *k* = 1, 2,…, generating function *C*(*z*) and first two moments *m*_1_ and *m*_2_. It is assumed that a burst in an arriving batch is allowed to join the system with probability *θ* depending upon the size of the burst. Consequently, *a*_*n*_, the probability for a group of *n* number of bursts to join the system from the arriving batch* of k* bursts, is given by *a*_*n*_. Consider

(2) $$\begin{array}{c} a_{n}=\left\{\begin{array}{cc} \overset{\infty }{\underset{k=1}{\sum }}C_{k}{\left(1-\theta \right)}^{k}, & n=0\\ \overset{\infty }{\underset{k=n}{\sum }}C_{k}\left(\begin{array}{c} k\\ n \end{array}\right)\theta^{n}{\left(1-\theta \right)}^{k-n}, & n\geq 1. \end{array}\right. \end{array}$$

The relationship between the probability generating functions of the sequences {*a*_*n*_} and {*c*_*k*_} is

(3) $$\begin{array}{c} a\left(z\right)=C\left(\theta z+1-\theta \right),\\ C\left(z\right)=\overset{\infty }{\underset{k=1}{\sum }}C_{k}Z^{k} \end{array}$$

and the corresponding moments satisfy the equation ${\overset{-}{a}}_{i}=\theta^{i}m_{i}$,  *i* = 1, 2. If the server is free then one of the admitted bursts enters the source and others join the buffer. Otherwise, all the admitted bursts enter the buffer and are retransmitted after some time with rate *η*. The retransmission time is exponentially distributed.

**Figure 3:**
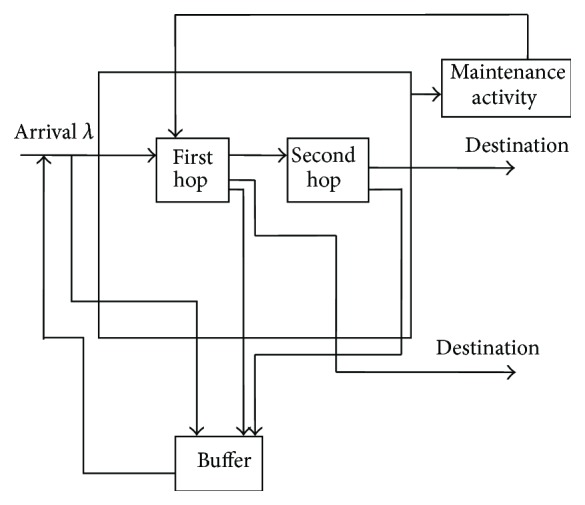
Block diagram of buffering and retransmission scheme.

The server provides first hop to all the bursts. As soon as the first hop is completed the burst may go for second hop with probability *β* or leave the system with probability *δ* or join the buffer for retransmission with probability *α*( = 1 − *β* − *δ*). After second hop is completed the burst may leave the system with probability *q* or join the buffer with probability *p*( = 1 − *q*) and the server may go for maintenance activity with probability *τ* or remain idle with probability $\overset{-}{\tau }=1-\tau$. The service times and maintenance activity times follow exponential distribution. The diagrammatic representation of the proposed method is given in Figure 3.

In this scenario, the retransmission, buffer, controllable arrival, first hop, second hop, contention, and maintenance activity correspond to the retrial, orbit, admission control, essential service, optional service, feedback, and vacation policy, respectively, in queuing terminology.

The first hop, second hop, and maintenance activity times are assumed to be exponentially distributed with respective rates *µ*_1_, *µ*_2_, and *γ*. Here *ρ*_1_ = *λ*/*µ*_1_, *ρ*_2_ = 2*λ* ^2^/*µ*_1_^2^, *ρ*_3_ = *λ*/*µ*_2_, *ρ*_4_ = 2*λ* ^2^/*µ*_2_^2^, *ρ*_5_ = *λ*/*γ*, and *ρ*_6_ = 2*λ* ^2^/*γ* ^2^.

(i) The probability that the server is idle in the empty system is
  (4) $$\begin{array}{c} I_{0}=\frac{\delta +\beta q-\left(\lambda /\lambda_{1}\right)\left(1-\omega \right)\theta m_{1}-\theta m_{1}\left(\rho_{1}+\beta \rho_{3}+\beta \tau \rho_{5}\right)}{\left(\delta +\beta q\right)\omega }, \end{array}$$
 where *ω* = *η*/(*η* + *λ*_1_) and *λ*_1_ = *λ*(1 − *a*_0_).
(ii) The probability that the server is idle in the nonempty system is given by
  (5) $$\begin{array}{c} I=I_{0}\left(1-\omega \right)\frac{\left[\left(\lambda /\lambda_{1}\right)\theta m_{1}-\delta -\beta q+\theta m_{1}\left(\rho_{1}+\beta \rho_{3}+\beta \tau \rho_{5}\right)\right]}{\delta +\beta q-\left(\lambda /\lambda_{1}\right)\left(1-\omega \right)\theta m_{1}-\theta m_{1}\left(\rho_{1}+\beta \rho_{3}+\beta \tau \rho_{5}\right)}. \end{array}$$
(iii) The probability that the server is busy providing first hop is
  (6) $$\begin{array}{c} P=\frac{\theta m_{1}\rho_{1}}{\left(\delta +\beta q\right)}. \end{array}$$

(iv) The probability that the server is busy providing second hop is
  (7) $$\begin{array}{c} Q=\frac{\beta \theta m_{1}\rho_{3}}{\left(\delta +\beta q\right)}. \end{array}$$
(v) The probability that the server is on maintenance activity is
  (8) $$\begin{array}{c} V=\frac{\beta \tau \theta m_{1}\rho_{5}}{\left(\delta +\beta q\right)}. \end{array}$$
(vi) Mean number of bursts in the buffer *L*_*q*_ is given by
  (9) $$\begin{array}{c} L_{q}=\frac{\left(AB-CD\right)}{{2B}^{2}}, \end{array}$$
 where
  (10) $$\begin{array}{c} A=-2I_{0}\omega \left[\alpha \theta m_{1}\rho_{1}+p\beta \left(\theta m_{1}\left(\rho_{1}+\rho_{3}\right)\right)\right]\\ B=\delta +\beta q-\frac{\lambda }{\lambda_{1}}\left(1-\omega \right)\theta m_{1}-\theta m_{1}\left(\rho_{1}+\beta \rho_{3}+\beta \tau \rho_{5}\right)\\ C=I_{0}\omega \left(\delta +\beta q\right),\\ D=\left(\frac{\lambda }{\lambda_{1}}\right)\left(1-\omega \right)\theta^{2}m_{2}-2\left(\frac{\lambda \theta m_{1}}{\lambda_{1}}\left(1-\omega \right)\left(\theta m_{1}\left(\rho_{1}+\beta \rho_{3}+\tau \beta \rho_{5}\right)+\alpha +p\beta \right)\right)-\theta^{2}m_{2}\rho_{1}-\theta^{2}m_{1}^{2}\rho_{2}-2\alpha \theta m_{1}\rho_{1}-\beta \left[\tau \left(\theta^{2}m_{2}\rho_{5}+\theta^{2}m_{1}^{2}\rho_{6}\right)+\theta^{2}m_{2}\rho_{3}+\theta^{2}m_{1}^{2}\rho_{4}\right]-2\left[\theta^{2}m_{1}^{2}\left(\tau {\rho_{1}\rho }_{5}+\tau {\rho_{3}\rho }_{5}+{\rho_{1}\rho }_{3}\right)+p\beta \theta m_{1}\left(\rho_{1}+\rho_{3}+\tau \rho_{5}\right)\right]. \end{array}$$

The mean number of bursts in the system *L*_*s*_ under steady state is

(11) $$\begin{array}{c} L_{s}=L_{q}+P+Q. \end{array}$$

From the above analysis, the number of bursts available in the buffer and in the system is calculated. Also various performance measures related with server in the network are calculated. In the simulation, the number of bursts being processed in the system under controllable and uncontrollable arrival is measured and compared.

## 3. Simulation and Discussion

**Figure 4:**
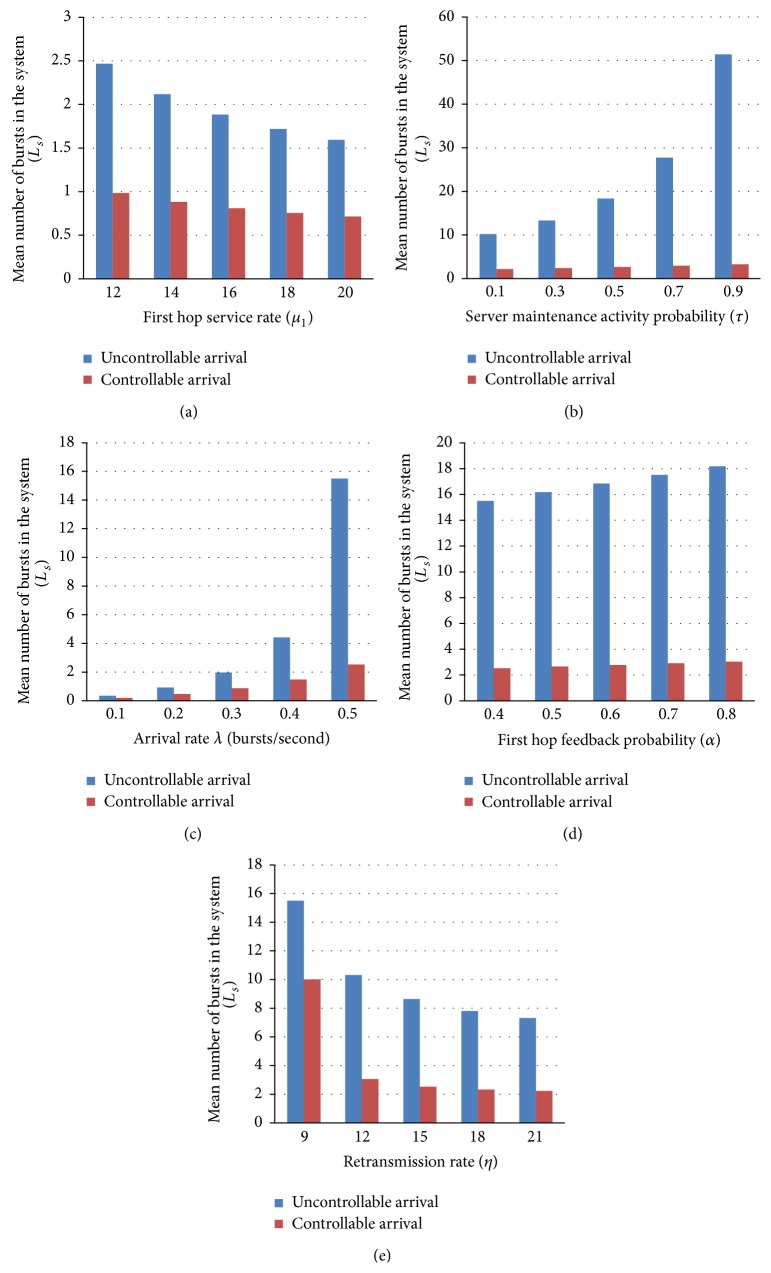
(a) First hop service rate versus mean number of bursts in the system. (b) Server maintenance activity probability versus mean number of bursts in the system. (c) Arrival rate versus mean number of bursts in the system. (d) First hop feedback probability versus mean number of bursts in the system. (e) Retransmission rate versus mean number of bursts in the system.

Numerical results are presented to study the effect of the arrival rate *λ*, first hop service rate *µ*_1_, maintenance activity probability rate *τ*, first hop feedback probability *α*, retransmission rate *η*, and admission control probability *θ* on various system characteristics. Here the default parameters are taken as *λ* = 0.5, *µ*_1_ = 5, *µ*_2_ = 4, *δ* = 0.3, *α* = 0.4, *p* = *q* = 0.5, *τ* = 0.4, *θ* = 0.7, *η* = 9, and *γ* = 5. Numerical results are compared for controllable and uncontrollable arrival and presented in Figure 4.

### 3.1. Simulation Results

Figures 4(a), 4(b), 4(c), 4(d), and 4(e) show the dependence of the first hop service rate *µ*_1_, server maintenance activity probability *τ*, the arrival rate *λ*, first hop feedback probability *α*, and retransmission rate *η* on the probability of mean number of bursts processed in the controllable and uncontrollable arrival system.

Figure 4(a) shows mean number of bursts processed in the system against first hop service rate for controllable and uncontrollable arrival. Mean number of bursts processed in the system decreases for the increase in service rate.  Figure 4(b) gives the impact of server maintenance activity probability for mean number of bursts processed in the system. If server maintenance activity probability increases, mean number of bursts processed in the system increases. Figures 4(c) and 4(d) give the relationship that increases in arrival rate of bursts and first hop feedback probability increases the mean number of bursts processed in the system. As retransmission rate increases number of bursts in the system decreases as shown in Figure 4(e).

## 4. Conclusion

In this paper, a hybrid buffering and retransmission scheme for Optical Burst Switching networks is analyzed with controllable arrival. Normally all the bursts reach the first hop and few of them go for second hop to reach destination. After each bursts reach the destination, the server may go for maintenance activity or wait for the arrival of next burst. Extensive simulation is done using MATLAB. Simulation results show how the arrival rate, service rate, retransmission rate, feedback probability, maintenance activity probability, and admission control affect the number of bursts processed in the system. In future work we plan to investigate the performance of the system by including impatient bursts and link failure in the system.
